# *In Vitro* Screening of Three Indian Medicinal Plants for Their Phytochemicals, Anticholinesterase, Antiglucosidase, Antioxidant, and Neuroprotective Effects

**DOI:** 10.1155/2017/5140506

**Published:** 2017-10-24

**Authors:** Mohan Penumala, Raveendra Babu Zinka, Jeelan Basha Shaik, Damu Amooru Gangaiah

**Affiliations:** Department of Chemistry, Yogi Vemana University, Kadapa, Andhra Pradesh, India

## Abstract

Cooccurrence of Diabetes Mellitus and Alzheimer's disease in elder people prompts scientists to develop multitarget agents that combat causes and symptoms of both diseases simultaneously. In line with this modern paradigm and as a follow-up to our previous studies, the present study is designed to investigate the crude methanolic extracts and subsequent CHCl_3_, *n*-BuOH, and H_2_O fractions of* Acalypha alnifolia*,* Pavetta indica,* and* Ochna obtusata* for their inhibitory activities towards specific targets involved in AD and DM, namely, acetylcholinesterase, butyrylcholinesterase, and *α*-glucosidase (*α*-Glc). The methanolic extract and its derived chloroform fractions exhibited remarkable inhibitory capacities with IC_50_ values being found at the *μ*g/mL level. Further studies on most active chloroform fractions presented a prominent ability to scavenge DPPH and ABTS reactive species and highest neuroprotective effect against H_2_O_2_ induced cell injury. Phytochemical analysis showed a large amount of phenolics, flavonoids, and terpenoids in active fractions. In conclusion,* A. alnifolia*,* P. indica,* and* O. obtusata* could be promising sources for the treatment of AD and DM since these fractions induced significant anticholinesterase, antidiabetic, antioxidant, and neuroprotection effects attributable to phenolic, flavonoid, and terpenoid contents and encourage further studies for development of multifunctional therapeutic agent for AD and DM dual therapy.

## 1. Introduction

Diabetes Mellitus (DM) is a complex, age-related metabolic syndrome that results from chronic hyperglycemia along with disturbances in carbohydrate, protein, and fat metabolism due to insulin deficiency, insulin resistance, or both. Being considered as a third “killer” of humankind, DM is affecting about 10% population of both sexes in all age groups all over the world [1]. Currently there were 366 million of individuals with DM worldwide, of whom about 80% are from developing countries. The scenario is expected to worsen in the next 20 years and reach 552 million by 2030 [2]. The chronic elevation of serum glucose in DM called hyperglycemia ultimately leads macro- and microvascular complications that accelerate ageing in organ systems. This results in damage of several tissues including retina, kidney, nerves, and blood vessels [3]. Therapies designed to reverse the chronic hyperglycemia in DM in a noninvasive manner are mostly based on inhibition of intestinal absorption of sugar. Hydrolysis of carbohydrates to monosaccharide by *α*-glucosidase (*α*-Glc) consequently elevates the absorbable glucose levels. This can be effectively controlled by inhibition of *α*-glucosidase and thus regarded as most prominent approach to both prevent and treat DM through improvement of hyperglycaemia [4–7].

Due to drastic improvement in the prevention and diagnostic strategies for DM complications, people are living longer with DM, which led to the emergence of new complications in recent times. Dementia is one example of those complications. Compared with the common people, the highest peak of incidence of dementia is 50–150% in people with DM [8]. According to Prince and coworkers, dementia is afflicting more than 35 million of individuals worldwide and expected to reach 115.4 million in the next two decades. Reflecting the world population ageing and continuous rise in prevalence of DM, the scenario is predicted to worsen in the coming days, if the association between these two age-related chronic disorders, namely, dementia and DM, is not discovered properly. Alzheimer's disease (AD) stands out as the most common form of dementia, accounting for 60–80% of all cases. Numerous epidemiological studies have shown that metabolic disturbances like hyperglycemia, hyperinsulinaemia, hypercholesterolaemia, and so forth associated with the DM are recognized to be linked with brain atrophy as well as the pathological hallmarks of AD [9].

AD that affects memory, thinking ability, and behavior is characterized by an insidious onset and a chronic progression with complex neuropathological features. The main pathological hallmarks of AD are formation of neurofibrillary tangles and senile plaques composed of amyloid *β* (A*β*) protein which initiates the inflammatory process, as well as the production of reactive oxygen species (ROS) and oxidative injury [10–12]. The cholinergic system is the most dramatically affected in AD brains, due to remarkable depletion of acetylcholine (ACh) levels. Concerning the cholinergic hypothesis, one of the rational and effective approaches to treat the AD's symptoms is raising the ACh through inhibition of acetylcholinesterase (AChE) that is responsible for hydrolysis of ACh. AChE inhibitors also have therapeutic applications in the treatment of Parkinson's disease, senile dementia, and ataxia. Furthermore, butyrylcholinesterase (BuChE), the second member of the cholinestrases family seems to be involved in the hydrolysis of ACh during the last stages of the disease in compensation for the reduced levels of AChE. Moreover, AChE and BuChE have been found to be responsible for upregulating the expression of the amyloid precursor protein (APP). Thereby dual inhibition of AChE and BuChE could be effective in the management of AD symptoms as it would result in the increase of ACh synaptic levels and the decrease of neurotoxic A*β* fibrils. Indeed, in damaged brain, preserving the AChE/BuChE activity ratio is essential for successful treatment of AD [13–15].

In recent years, more and more attention is paid to oxidative stress as it associated with acute and chronic diseases like AD and DM. This is strongly supported by validated biomarkers of oxidative stress such as lipid peroxidation, modification of DNA, protein oxidation, and ROS formation in these diseases. Numerous studies have also suggested that oxidative stress and A*β* protein are linked to each other. Therefore, antioxidant therapies by scavenging free radicals are considered as a potential means for the prevention or the treatment of AD and DM [16].

The few agents approved by the US Federal Drug Administration (FDA) for the treatment of AD and DM have less potency and multiple side effects [12]. Consequently, it has become a necessity to develop the new agents that are pharmacologically safe, cost effective, and immediately available with minimal side effects. In this connection, the World Health Organization has also recommended the development of improved and safer herbal medicines in this concern.

Since time immoral, medicinal plants have been recognized as important to the global economy as they contribute approximately 85% of traditional medicine preparations. In addition, medicinal plants naturally are a rich source of secondary metabolites with various beneficial effects on human health. Thus, medicinal plants are nowadays regarded as valuable material for development of modern medicines, nutraceuticals, food supplements, pharmaceutical intermediates, and chemical entities for synthetic drugs [17]. By contrast, due to less effort towards examining extracts of plants, scientific information on medicinal properties of various plants is still scarce. Realizing their importance, it is highly essential to explore the extracts of medicinal plants extensively for different bioactivities. In this context, some years ago we embarked on a long-term research project aimed at the development of potential multifunctional agents for dual therapy of age-related and associated diseases AD and DM. As part of this project, we report herein the extraction and fractionation of* Acalypha alnifolia* Klein ex Willd.,* Pavetta indica* L., and* Ochna obtusata* DC. and evaluation of their inhibitory activities against AChE, BuChE, and *α*-Glc target enzymes involved in AD and DM. Kinetic analysis of inhibition, DPPH and ABTS radical scavenging activities, neurotoxicity, and neuroprotective ability against H_2_O_2_ induced cell death in human neuroblastoma cell lines and phytochemical analysis of most active fractions were also described.

## 2. Material and Methods

### 2.1. Chemical Reagents and Enzymes

Common chemicals used in this study were of reagent grade or better and used without further purification.* Ee*Acetylcholinesterase (EC 3.1.1.7, from Electric eel),* Eq*butyrylcholinesterase (EC 3.1.1.8, from Horse serum), 2,2′-azino-bis(3-ethylbenzthiazoline-6-sulfonic acid) (ABTS), 2,2-diphenyl-1-picrylhydrazyl radical (DPPH), 5,5′-dithio-bis(2-nitrobenzoic acid) (DTNB),* p*-nitrophenyl *α*-D-glucopyranoside and *α*-glucosidase (EC 3.2.1.20 from* Saccharomyces cerevisiae*), acetylthiocholine iodide, butyrylthiocholine iodide, 3-(4,5-dimethylthiozol-2-yl)-2,5-diphenyltetrazoliumbromide (MTT), Folin-Ciocalteu reagent, galantamine, and trolox were purchased from Sigma-Aldrich (St. Louis, MO, USA). SK N SH human neuroblastoma cells were obtained from National Centre for Cell Sciences (Pune, India). All biochemicals and buffer chemicals were purchased from Merck.

### 2.2. Collection of Plant Material

Based on the ethnopharmacological records, the titled plants were selected (Table 1). The plant materials were collected from Lankamalla forest in Kadapa district, Andhra Pradesh, India, during October 2013. A. Madhusudana Reddy, Assistant Professor, Department of Botany, Yogi Vemana University, identified the harvested materials employed and voucher specimens vide numbers YVU02 AGD, YVU54 AGD, and YVU61 AGD were deposited in the herbarium of Yogi Vemana University. The plant materials were air-dried at 25–30°C for two weeks, weighed, ground, and sieved into fine powder. The smooth powder was stored in an air-tight container and kept in darkness at −20°C until further use.

### 2.3. Plant Extraction and Fractionation

For extract preparation, ground plant material (150 g each) was extracted twice with 500 mL of 90% methanol by soaking at 35°C for 48 h. The plant extracts were filtered through Whatman Number 1 filter paper. The combined filtrates were concentrated using rotary evaporator (Heidolph, Germany) under reduced pressure at 30°C. The crude methanolic extracts were suspended in water (50 mL) and chloroform (100 mL) was added and shaken well, and the layers were allowed to separate for 6 h in a separating funnel. Chloroform layer was separated and evaporated to obtain the chloroform fraction. Similar protocol was repeated with *n*-butanol. Each of the fractions obtained was dried using Rotavapor. The remaining water solubles were concentrated under reduced pressure to get water fraction. Percentage yields of extracts and fractions were calculated and expressed in terms of air dried weight of plant material.

### 2.4. Cholinesterase Enzyme Inhibitory Activity

The methanolic extracts of three plants (AAM, PIM, and OOM) and their derived fractions (AAC, AAB, and AAW; PIC, PIB, and PIW; OOC, OOB, and OOW) were screened for their inhibitory activity against AChE and BuChE enzymes using Ellman's colorimetric method [25]. The AChE and BuChE inhibition assay were carried out spectrophotometrically for methanolic extracts and their derived fractions using acetylthiocholine iodide (ATCI) and butyrylthiocholine iodide (BTCI), respectively, as substrate. In a 96-well plate, 10 *μ*L of enzyme (AChE, 2 U/mL, and BuChE, 2 U/mL), 10 *µ*L of plant extract or fraction (15, 30, 90, and 150 *μ*g/mL), and 100 *µ*L phosphate buffer were taken. To this mixture, 50 *µ*L of DTNB solution (3.96 mg of DTNB and 1.5 mg of sodium bicarbonate dissolved in 10 mL of 200 mM phosphate buffer pH 7.7) was added and incubated for 5 minutes at 25°C. Substrates with the volume of 15 *μ*L (10.85 mg of ATCI or BTCI in 5 mL phosphate buffer) were added and total mixtures were incubated for a further 5 minutes at 25°C. The yellow color developed due to the formation of an anion 5-thio-2-nitrobenzoate by the reaction between thiocholines and DTNB was measured at 412 nm. Galantamine, with different concentrations (0.12, 0.23, 0.46, 0.92, 1.84, 3.68, and 7.37 *μ*g/mL) and the test mixture containing all the components except plant sample were taken as positive and negative controls, respectively. The percent of inhibition was determined by comparing the reaction rates for the sample to the negative control. The IC_50_ values were determined graphically from inhibition curves plotting log inhibitor concentration versus percent of inhibition.

### 2.5. *α*-Glucosidase Inhibitory Activity

The inhibition of *α*-glucosidase activity by methanolic extracts and its derived fractions was assayed with the modified method of Shibano et al. using* p*-nitrophenyl *α*-D-glucopyranoside as substrate [26]. The assay solution with 10 *μ*L extract or fraction at various concentrations (15, 30, 90 and 150 *μ*g/mL), 100 *μ*L phosphate buffer pH 6.8 and 50 *μ*L of enzyme (*α*-glucosidase, 0.15 unit/mL) was incubated at 37°C for 15 min. Substrate with a volume of 50 *μ*L (0.5 mM* p*-nitrophenyl *α*-D-glucopyranoside) was added and incubated further at 37°C for 15 min. To this, added 50 *μ*L of 200 mM Na_2_CO_3_ to terminate the reaction. Enzyme activity was determined spectrophotometrically at 415 nm through measuring the quantity of* p*-nitrophenol released. The percent of inhibition and IC_50_ values were determined.

### 2.6. Kinetic Study on Enzyme Inhibition

To predict the mode of inhibition and inhibition constants, reciprocal plots of 1/*V* versus 1/[S] were constructed at different concentrations of the substrate of different enzymes by using method mentioned in the activity assay on the basis of enzyme [27]. The assay mixture (250 *μ*L) consists of 145 *μ*L of 200 mM phosphate buffer (pH 7.7), 80 *μ*L of DTNB (18.5 mg of DTNB dissolved in 10 mL phosphate buffer pH 7.7), and 10 *μ*L of enzyme and 15 *μ*L of substrate (10, 25, 50, and 100 *μ*M). Four different concentrations of inhibitors (15, 30, 90, and 150 *μ*g/mL) were added to the assay solution and preincubated for 5 min at 25°C with the addition of substrate in different concentrations. The parallel control experiments were performed without inhibitor in the assay.

Progress curves were constructed at 412 nm over 5 min. Then, double reciprocal plots (1/*v* versus 1/[s]) were constructed using GraphPad Prism version 5.0. The replots of the slopes and intercepts of the double reciprocal plots against inhibitor (extract) concentrations gave the inhibitor constants (*K*_*i*1_ for binding to free enzyme and *K*_*i*2_ for enzyme-substrate complex) as the intercepts on the *x*-axis. Data analysis was performed using Microsoft Excel.

### 2.7. Antioxidant Activity Assay

#### 2.7.1. ABTS Free Radical Scavenging Assay

ABTS (2,2'-azinobis [3-ethylbenzthiazoline]-6-sulfonic acid) radical scavenging activity was measured by the method of Re et al. [28] with some modifications. The solutions of 2 mM ABTS and 2.45 mM potassium persulfate were prepared and thoroughly mixed. Then, the solution was kept in dark at room temperature throughout the night to produce free radicals. Finally, 1 mL of ABTS radical solution was added to 3 mL of pyrogallol solution in ethanol at different concentration range of 10–30 mg/mL. The assay mixture was incubated in the dark for 30 min. After addition of 10 mL of test solution, the remaining ABTS is determined at 745 nm on a spectrophotometer. The calibration curve was prepared with trolox as a positive control and results were expressed in trolox equivalents. A plot of trolox concentration with % ABTS scavenging activity was used as the standard curve. Based on this, the concentrations of plant fractions were expressed as micromole equivalent of trolox *µ*mol/g.

#### 2.7.2. DPPH Radical Scavenging Activity

DPPH radical scavenging potential of active chloroform fraction of methanolic extract of titled plants was estimated according to modified assay as described by Sarikurkcu et al. [29]. Chloroform fraction (1.5 mL) of methanolic extract of each plant at different concentrations (50, 100, 200, and 400 *μ*g/mL) was added to 9 mL of the DPPH solution (60 mM). The reaction mixtures were prepared under dim light. After vigorous shaking, the mixtures were incubated for 30 min in the dark. The decrease in the purple coloration of the reaction mixtures due to bleaching of the DPPH color was measured at 517 nm using 96-well microplate reader. Ascorbic acid was used as a positive control and methanol as a negative control. The calibration curve was prepared with ascorbic acid as positive control and results were expressed in micromole ascorbic acid equivalents.

### 2.8. Cell Culture and Treatment

The SK N SH, human neuroblastoma cells were cultured in MEM containing 0.5 mM L-glutamine, 0.1 mM sodium pyruvate, and 1 mM nonessential amino acids with 10% FBS in a highly humidified atmosphere having 5% CO_2_ at 37°C. Cell cultures at approximately 80% confluence were used for all* in vitro* experimental procedures.

#### 2.8.1. Measurement of Cell Viability by MTT Assay

Cytotoxic effect of selected plant fractions on the cell viability was measured using MTT assay as described in our previous studies [27]. SK N SH cells (0.2 × 10^6^ cells per well) in 200 mL of corresponding medium with 10% FBS were seeded into 96-wellplate. Increasing concentrations of chloroform fractions (50, 100, 200, and 400 *µ*g/mL) dissolved in DMSO were added to the cells and incubated at 37°C for 24 h in a humidified CO_2_ incubator with 5% CO_2_. The medium was replaced along with 20 *μ*L of 5 mg/mL MTT. It was further incubated for 4 h in humidified atmosphere, the medium was removed, and 200 mL of 0.1 N acidic isopropyl alcohol was added to the wells to dissolve the MTT formazan crystals. Absorbance was recorded at 570 nm immediately after the development of purple color. Relative cell viability was evaluated according to the quantity of MTT converted into insoluble formazan salt. The optical density of the formazan generated in the control cells was considered to represent 100% viability. The results were expressed as mean percent of viable cells versus respective control.

#### 2.8.2. Protection against H_2_O_2_ Induced Cell Death in SK N SH Cells

Neuroprotective effect of selected plant fractions against H_2_O_2_ induced oxidative injury in SK N SH cells was determined [26]. Thus, SK N SH cells were pretreated with different concentrations of fractions (15–150 *μ*g) for 3 h before treatment with H_2_O_2_ (1.0 mM). H_2_O_2_ was added to the medium to induce oxidative stress in SK N SH cell and incubated for 24 h in humidified atmosphere. The percent of cell viability was measured by MTT colorimetry as described above.

### 2.9. Phytochemical Analysis

#### 2.9.1. Determination of Total Phenolic Contents

Total phenolic contents (TPCs) were estimated by Folin-Ciocalteu (FC) reagent method with slight modifications [30]. 35 *μ*L each of the 5 mg/mL of chloroform fraction was added to 150 *μ*L of Folin-Ciocalteu reagent (1 mg Folin-Ciocalteu in 10 mL of distilled water) and incubated for 5 min. Then, 115 *μ*L of 7.5% Na_2_CO_3_ was added to the reaction mixture. The resulting mixture was incubated for the next 30 min at 45°C followed by 60 min incubation in room temperature, and absorbance was measured at 765 nm using a spectrophotometer. Entire setup should be kept in dark condition. Standards of gallic acid in the concentration range 0 to 100 *μ*g/mL were run with the test samples. Total phenols were determined using the standard curve obtained for gallic acid and the results were expressed as mg of gallic acid equivalents (mg GAE/g) per g of dry plant part.

#### 2.9.2. Determination of Total Flavonoid Contents

The total flavonoid content was determined according to the aluminum chloride colorimetric method [31, 32]. Briefly, 0.5 mL of chloroform fraction was mixed with mixture of 1.5 mL of 95% alcohol, 0.1 mL of 10% aluminum chloride hexahydrate (AlCl_3_·6H_2_O), 0.1 mL of 1 M potassium acetate (CH_3_COOK), and 2.8 mL of deionized water. After incubation at room temperature for 40 min, the reaction solution absorbance was measured at 415 nm by using a spectrophotometer against a blank which has all the reaction mixture except plant fraction. The total flavonoid content was calculated using a standard curve of rutin and expressed as mg of rutin equivalents (mg RE/g).

#### 2.9.3. Determination of Total Tannins Contents

Total tannins were counted by Vanillin HCl method [33]. To 1 mL of chloroform fraction, 3 mL of acidic methanol was added and the mixture was incubated at room temperature for 10 min and, then, 6 mL of Vanillin HCl reagent was added. Absorbance was observed at 500 nm. Catechin was chosen as the standard reference and values were expressed as mg of Catechin equivalents per mL (CE/mL).

#### 2.9.4. Determination of Total Terpenoids

Total terpenoids were estimated using an assay developed by Ghorai et al. [34]. To the 160 *µ*L of fraction, 1.2 mL of chloroform was added. The sample solution was vortexed completely and allowed for 3 min to rest. 100 *µ*L conc. H_2_SO_4_ was added in the test solution by using ice both slowly and not more than 15 min. Then the assay tube was incubated at room temperature for 1.5–2 h in dark. At the end of incubation time a reddish brown precipitate was formed. Then supernatant of the reaction mixture was decanted carefully and gently without disturbing the precipitate. 1.5 mL of 95% (Vol/Vol) methanol was added to the precipitate and vortex thoroughly until all the precipitate dissolves in methanol completely. Absorbance was read at 538 nm against a respective solvent used for extraction.

#### 2.9.5. Total Alkaloid Content

Total alkaloids were estimated by using spectrophotometric method based on the reaction with BCG with appropriate changes [35]. Briefly, BCG solution was prepared by heating 69.8 mg BCG with 3 mL of 2 N NaOH and 5 mL distilled water until being completely dissolved and the solution was diluted to 1000 mL with distilled water. Boldine standard solution was made in HCl (pH 2.5) at concentration of 1 mg per mL. For the preparation of standard curve, accurately measured aliquots of boldine standard solutions were transferred to each different separatory funnels. Then, 5 mL of phosphate buffer pH 4.7 was added before 5 mL of BCG solution and shaken vigorously. Furthermore, 5 mL of chloroform was added and after shaking, and a yellow-colored complex with high absorption containing boldine-BCG solution was produced. The absorbance was measured at 470 nm against blank.

### 2.10. Statistical Analysis

All the tests were performed in triplicate and data were expressed as means ± Standard Error of Mean (SEM). Percentage inhibition values were log-transformed before being subjected to statistical analysis. Statistical comparison were performed by one-way analysis of variance (ANOVA) using SPSS version 10 software (SPSS Inc., Chicago, USA) and the values were considered as statistically significant when *p* values were less than 0.05 (*p* < 0.05). The IC_50_ values were calculated from the logarithmic nonlinear regression curve derived from the plotted data using GraphPad Prism software version 5.0 (GraphPad Software, Inc., San Diego, USA).

## 3. Results and Discussion

### 3.1. Extraction Yields

Efficient extraction methods were proved to be a crucial step for getting extracts with acceptable yields. The powdered plant material was extracted with 90% methanol in water and the obtained crude extract was fractionated successively to yield chloroform, *n*-butanol, and water solubles. Percentage yields of the methanol extract and its derived fractions were showed in Table 2. Data showed the different extractive capacities of each solvent. The extraction yield of titled plants with 90% methanol came in the range from 15.34 to 17.68 with the highest extractive capacity. Extraction yield of different fractions was obtained in descending order of residual aqueous > chloroform >  *n*-butanol.

### 3.2. *In Vitro* Biological Activity

To the best of our knowledge, this is the first report to assess methanolic extract and various derived fractions of* A. alnifolia*,* P. indica,* and* O. obtusata* for their multifunctional potency against both AD and DM. Therefore, a reliable evaluation protocol requires different activity evaluations linked to pathophysiological targets to account for various mechanisms of anti-AD and anti-DM action. In the present study, several* in vitro* assays on relevant targets of AD and DM have been used to determine multifunctional ability of titled plants.

#### 3.2.1. Cholinesterase Inhibitory Activity

**Figure 1:**
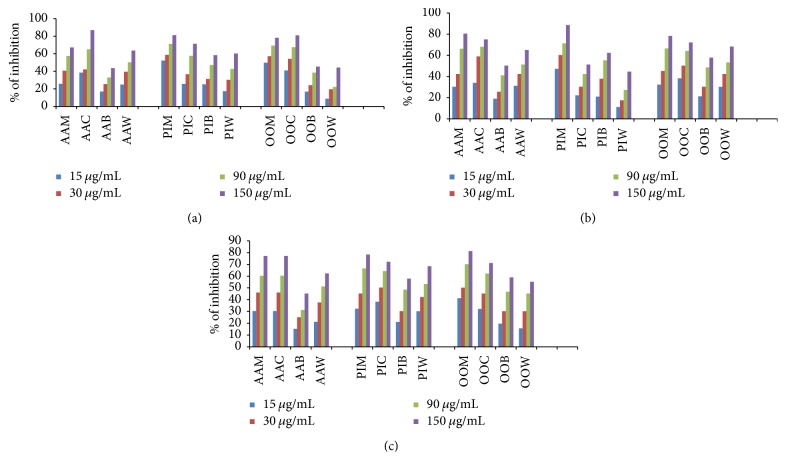
Dose-dependent percent inhibition by plant methanolic extract and its derived fractions on AChE (a); BuChE (b); *α*-Glc activity (c); AAM, PIM, and OOM: methanolic extracts of* A. alnifolia*,* P. indica,* and* O. obtusata*, respectively; AAC, PIC, and OOC: chloroform fractions of methanolic extract of* A. alnifolia*,* P. indica,* and* O. obtusata*, respectively; AAB, PIB, and OOB: *n*-butanol fractions of methanolic extract of* A. alnifolia*,* P. indica,* and* O. obtusata*, respectively; AAW, PIW, and OOW: water fraction of methanolic extracts of* A. alnifolia*,* P. indica, *and* O. obtusata*, respectively.

The methanolic extracts of three plants (AAM, PIM, and OOM) and their derived fractions (AAC, AAB, and AAW; PIC, PIB, and PIW; OOC, OOB, and OOW) were screened for AChE and BuChE inhibitory activities using Ellman's colorimetric method in 96-well microplate [36]. Galantamine was taken as a reference drug. In this assay, methanolic extract of all plants and their derived fractions at different concentrations (15, 30, 90, and 150 *μ*g/mL) showed dose-dependent inhibitory activity against enzymes AChE and BuChE (Figure 1). As tabulated in Table 3, the AAM, PIM, and OOM extracts were found to be potent in inhibiting AChE and BuChE enzyme activity with IC_50_ ranges from 17.77 ± 3.17 to 82.16 ± 2.55 *μ*g/mL. Thus, all these three plants can be considered as dual inhibitors of AChE and BuChE enzymes. Among all the fractions, AAC, PIC, and OOC showed the highest activity against AChE and BuChE enzymes with IC_50_ range of 19.83 ± 1.28 to 52.09 ± 2.5 *μ*g/mL [for IC_50_ graphs, see Supplementary Data, available online at https://doi.org/10.1155/2017/5140506]. 

As far as AChE is concerned, OOC is the most potent inhibitor with IC_50_ of 25.7 ± 0.3 *μ*g/mL followed by AAC and PIC. Fractions AAW, PIB, and PIW displayed significant inhibition on AChE activity. However, AAB, OOB, and OOW were less active against AChE with IC_50_ values more than 150 *μ*g/mL.

Regarding BuChE, similar pattern was observed in the inhibition activity. Fraction OOC was found to be the most potent in inhibition with IC_50_ of 19.83 ± 1.28 *μ*g/mL whereas AAC and PIC were almost equipotent. Fractions AAW and PIB displayed significant inhibition on BuChE activity. However, AAB, PIW, OOB, and OOW were less active.

#### 3.2.2. *α*-Glucosidase Inhibitory Activity

To assess the antidiabetic potency of titled plants, the extracts and derived fractions were tested for their *α*-glucosidase inhibitory activity by* in vitro* enzyme assay as reported by Shaik et al. [27] and the IC_50_ values are showed in Table 3. Acarbose was used as a reference drug. According to data, among the methanolic extracts, OOM showed significantly higher inhibitory activity with IC_50_ value of 28.7 ± 1.9 *μ*g/mL. The methanolic extracts AAM and PIM have shown almost similar potency against *α*-glucosidase with IC_50_ values of 45.24 ± 4.01 and 42.76 ± 2.0, respectively. Among fractions, as expected AAC, PIC, and OOC fractions inhibited the *α*-glucosidase activity potentially with IC_50_ values of 41.86 ± 1.31, 35.29 ± 1.6, and 39.3 ± 1.6, respectively. Additionally the *n*-butanol and water fractions of these three plants showed moderate and low inhibitory activity on *α*-glucosidase [for IC_50_ graphs, see Supplementary Data].

#### 3.2.3. Kinetics of Inhibition

**Figure 2:**
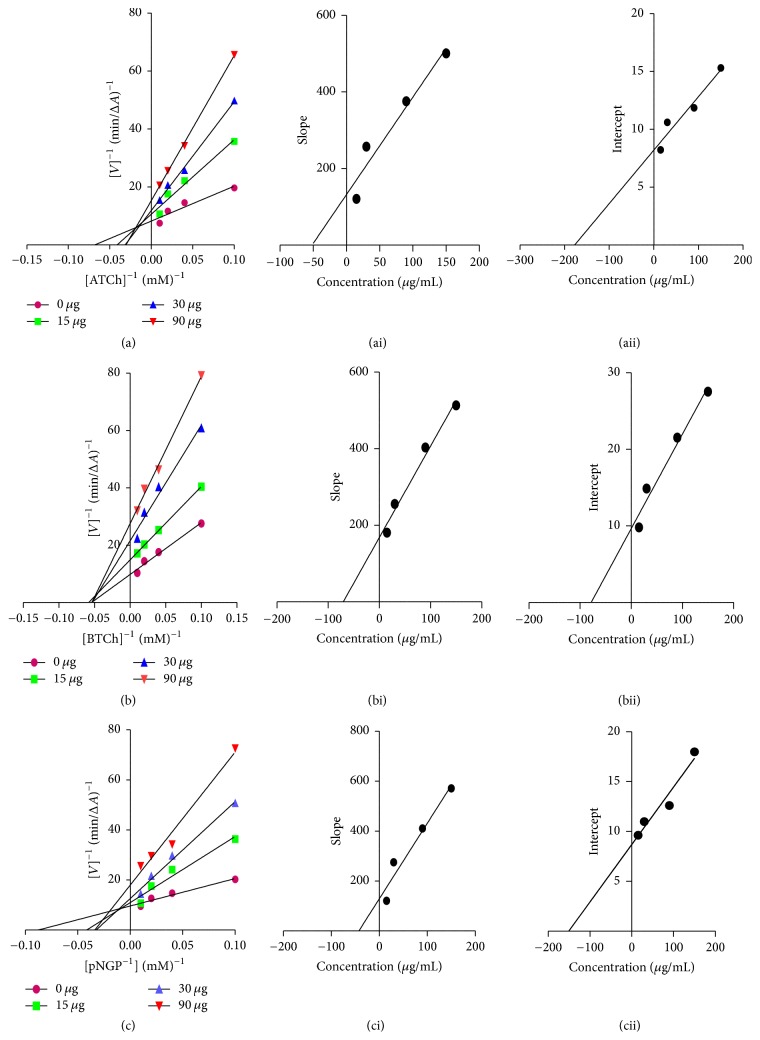
Steady-state inhibition of AChE (a), BuChE, (b) and *α*-Glc (c) by most active fraction (AAC) from* A. alnifolia*. (a–c) Lineweaver-Burk plot of reciprocal of initial velocities versus reciprocal of substrate concentrations (0.1–0.5 mM) in the absence and presence of AAC at 15, 30, and 90 *µ*g. (ai–ci, aii–cii) Secondary plots of the Lineweaver-Burk plot, slope versus various concentrations of AAC (i) regarding inhibition constant *K*_*i*1_ and intercept versus various concentrations of AAC (ii) regarding inhibition constant *K*_*i*2_.

Most active fractions AAC, PIC, and OOC were selected for kinetic study to unveil their mode of inhibition to enzymes. At first, the exact rate of enzyme activity was measured at four different concentrations of inhibitor using different concentrations of the corresponding substrate. In each case, the initial velocity was determined at different concentrations of the substrates (S), and the reciprocal of the initial velocity (1/*v*) was plotted against the reciprocal of concentrations of substrates. The mode of inhibition was determined by employing these double reciprocal Lineweaver-Burke plots where increased slopes and intercepts at increasing concentration of the inhibitor and intersecting data points at the upper left quadrant were noticed (Figure 2) [27]. This observation revealed a mixed type of inhibition for active fractions (AAC, PIC, and OOC) [see Supplementary Data]. The mixed inhibition is a midway of the competitive and uncompetitive inhibition. Mixed inhibitors were able to bind with either the free enzyme (E) or the enzyme-substrate complex (E + S). The two inhibition constants in the mixed inhibition, *K*_*i*1_ and *K*_*i*2_, signified the equilibrium constant of dissociation of the inhibitor-enzyme complex and the inhibitor-bound enzyme-substrate complex. These values for active fractions AAC, PIC, and OOC were computed from the secondary plots of the slope and *Y*-intercept from the Lineweaver-Burke plot versus inhibitor concentration and were showed in Table 4. These constants show that all active fractions have strong potency to bind to the free enzyme.

#### 3.2.4. Antioxidant Activity

Radical scavenging activities (RSA) play a very important role in preventing the deleterious effect of free radical in different diseases including AD and DM. The antioxidant activities of active fractions AAC, PIC, and OOC at various concentrations were determined using the free radicals DPPH and ABTS.

The ABTS assay is a widely accepted antioxidant assay to screen the total antioxidant power of fruits, vegetables, foods, and plants [28]. In particular, it is recommended to be used for plant extracts because the long wavelength absorption maximum at 745 nm eliminates color interference in plant extracts. In this assay, the ABTS radical cation is generated by the oxidation of ABTS with potassium persulfate, its reduction in the presence of hydrogen-donating antioxidants in chloroform fractions is measured spectrophotometrically, and results were expressed as micromole trolox equivalent antioxidant capacity (TEAC/g) as shown in Table 5. A higher TEAC value meant that the sample had a stronger antioxidant activity. Antioxidant activities of the chloroform fractions of methanolic extracts of the three tested plants were in the following decreasing order: OOC (171.006 ± 3.32 *µ*mol TE/g) > PIC (150.29 ± 1.58 *µ*mol TE/g) > AAC (143.39 ± 2.17 *µ*mol TE/g). The antioxidant capacities obtained from the ABTS assay were in good accordance with those reported in literature. Song et al. reported the radical scavenging activities of 56 selected Chinese medicinal plants against the ABTS radical and the values of antioxidant capacities were in the range of 0.61 to 708.73 *μ*mol TE/g [37]. Among 56 tested plants,* Dioscorea bulbifera* showed the highest antioxidant capacity (708.73 *μ*mol TE/g), followed by* Eriobotrya japonica* (326.87 *μ*mol TE/g), and* Tussilago farfara* (217.62 *μ*mol TE/g) which were significantly higher than our results. The TEAC values of* Ephedra sinica* (197.69 *μ*mol TE/g) and* Ardisia japonica* (164.17 *μ*mol TE/g) were almost in the same range of TEAC of our titled plants. On the contrary, the values (0.61 to 96.12 ± 2.20 *μ*mol TE/g) for most of the plants were significantly lower than our results.

The 2,2-diphenyl-1-picrylhydrazyl radical is a stable free radical that had an absorption band at 517 nm. When it accepts an electron from antioxidant, bleaching of purple-colored solution to yellow is visualized. The degree of color change is proportional to the concentration and potency of the antioxidants. A huge decrease in the absorbance indicates significant free radical scavenging activity of the extract under test [29]. All the three active chloroform fractions AAC, PIC, and OOC were tested for their DPPH radical scavenging capacity and the results was expressed as ascorbic acid equivalents (AAE/g) as demonstrated in Table 5. In this study, all three fractions were able to decolorize DPPH and the free radical scavenging potentials of the fractions were found to be in the order of PIC (204.4 ± 2.21 *µ*mol AAE/g) > OOC (183.96 ± 5.29 *µ*mol AAE/g) > AAC (127.18 ± 1.28 *µ*mol AAE/g). Vertuani et al. [38] obtained values ranging from 4.27 to 30.2 *µ*mol ascorbic acid equivalents/g for tea extracts. Gil et al. [39] reported the total antioxidants of fruit extracts (expressed as ascorbic acid equivalents) in the range from 0.403 to 5.03 *µ*mol/100 g as determined by the DPPH method. Compared with this literature, our results were in good agreement with the previous studies. Thus, this method represented PIC, OOC, and AAC as valuable antioxidant extracts. The logic behind this behavior may be explained by the presence of phytoconstituents that are capable of donating hydrogen or electron to a free radical to scavenge the potential damage by them.

#### 3.2.5. Cell Viability

**Figure 3:**
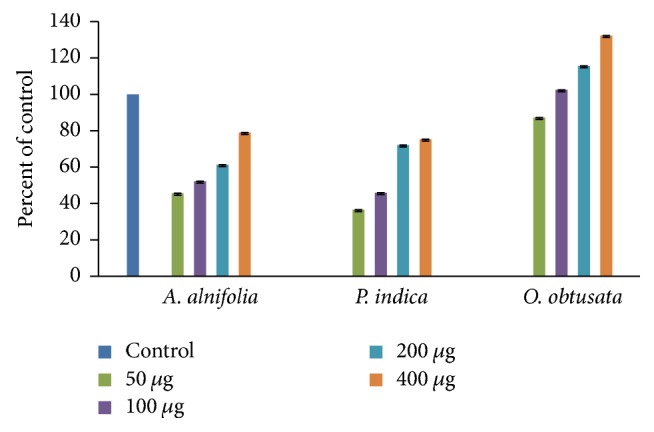
Neurotoxic effects of* A. alnifolia*,* P. indica,* and* O. obtusata* on SK N SH cells (human neuroblastoma cell line). Bar chart shows the percentage of cell viability in the presence or absence (control) of indicated concentrations of* A. alnifolia*,* P. indica,* and* O. obtusata*. MTT assay was done to assess cell viability. Cell viability corresponding to control cells was considered as 100%. The values represent the mean SEM of three independent experiments, each one performed in triplicate in different cell batches. Statistics show neurotoxic effects of these plant extracts versus controls. p < 0.05 versus 100% cell viability (one-way ANOVA test).

The safety of the extract is absolutely crucial for a successful pharmaceutical formulation. In line with this, the possible toxic effects of active fractions AAC, PIC, and OOC in the human neuroblastoma SK N SH cells have been assayed with MTT assay and results were summarized in Figure 3. After 24 h incubation of fractions AAC, PIC, and OOC at increasing concentration (50, 100, 200, and 400 *μ*g/mL) in culture medium, the cell viability was determined. The results as displayed in Figure 3, under the experimental conditions AAC, PIC, and OOC, displayed cell viability ranging from 36 up to 132%. Among the tested fractions, fraction OOC attenuated the cell toxicity significantly in a concentration dependent manner. Interestingly, the cell survival was increased with escalating cell proliferation even in the presence of high concentrations of OOC and was nontoxic to SK N SH cells. This observation suggests that certain compounds present in OOC were likely to promote cell survival or delay the natural death of neurons in culture medium [40, 41].

#### 3.2.6. Neuroprotective Capacities against H_2_O_2_ Induced Cell Death in SK N SH Cells

**Figure 4:**
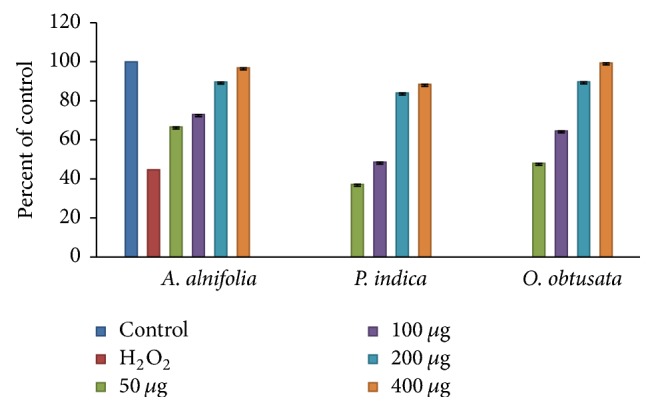
Neuroprotective activity of* A. alnifolia*,* P. indica,* and* O. obtusata* against H_2_O_2_ induced cell death in SK N SH cells. Cells were treated with different concentrations of 50–400 *µ*g of the AAC, PIC, and OOC for 3 h. After 24 h, cell viability was determined by MTT assay in the presence of 1.0 mM H_2_O_2_. The values represent the mean SEM of three independent experiments, each one performed in triplicate in different cell batches. Statistics show neurotoxic effects of these extracts versus controls. *p* < 0.05 versus H_2_O_2_ treatment.

The neuroprotective capacities of active fractions against oxidative stress were evaluated by assessing the ability of the fractions to protect against H_2_O_2_ induced SK N SH cell injury. For this purpose, the cell viability assay was performed by MTT innersalt determination. SK N SH viability was greatly reduced when exposed to H_2_O_2_ and the cytotoxicity of H_2_O_2_ was concentration and time dependent in MTT assay [27]. The survival rates of SK N SH cells were about 44.65% when the cells were treated with 1.0 mM of H_2_O_2_ for 8 h. The viability of SK N SH cells pretreated with active fractions AAC, PIC, and OOC at 50, 100, 200, and 400 *μ*g, 24 h before exposure to H_2_O_2_, was significantly increased in a dose-dependent manner (Figure 4). When the neuroprotective effect induced by fractions was compared with control, very interestingly, all fractions showed neuroprotectivity similar and equal to control at higher concentrations. This observation suggests that certain compounds present in fractions were likely to promote cell survival or delay the death of neurons in culture medium [41]. In all, and based on the results obtained, these fractions can be considered as potential oxidative suppressors.

### 3.3. Phytochemical Analysis

Compounds like phenolics, flavonoids, tannins, terpenoids, alkaloids, carotenoids, and so forth derived from plant sources have hopeful health promoting properties and protective effects against chronic diseases while acting in combination [35]. Thus, phytochemical analysis provides basic information about medicinal importance of a plant extract.

Phytochemical studies on the most active chloroform fractions of titled plants revealed the presence of phenolics, flavonoids, tannins, terpenoids, and alkaloids. Among the estimations, the total flavonoid and total phenolic content were significantly higher in all three active fractions AAC, PIC, and OOC. However, fraction OOC was found to be rich in terpenoid content. Total phenolic content (TPC) in the active fractions was investigated by the Folin-Ciocalteu method which is expressed as gallic acid equivalents (mg GAE/g extract). Aluminum-flavonoids complex formation assay was used for the quantification of total flavonoid content (TFC). The TFC was represented as rutin equivalents (mg RE/g extract). Total terpenoids were quantified with standard method and noted as linalool equivalents (mg LE/g extract).

Based on the data expressed in Table 6, OOC fraction has higher amounts of phenolic content (174.9 ± 7.6 mg GAE/g) and flavonoid (260.93 ± 22.4 mg RE/g) and total terpenoid contents (358.7 ± 0.9 mg LE/g). Least amounts of tannins and alkaloids were detected in all three fractions. Among the tested fractions, highest amount of alkaloids (16.85 ± 1.24 mg AE/g) were found in PIC. In the process of detecting tannins, OOC has higher quantity (14.658 ± 0.045 mg CE/g) compared to others.

Flavonoid derivatives were reported to have tremendous effects on human health covering a wide spectrum of biological activities such as antibacterial, antiviral, anti-inflammatory, anticancer, and antiallergic activities. Flavonoids have also shown to be highly effective scavengers of various free radicals and significantly effective in cholinesterase and glucosidase inhibition implicated in diseases AD and DM. Phenolic compounds were widely found to be responsible for the antioxidant activity of plant materials and hence many of the natural polyphenols possess therapeutic potential for AD and DM [42]. The terpenoids are abundant chemical constituents found in nature. Pharmacologically, terpenoids have been shown to have antibacterial, antifungal, antimalarial, anticarcinogenic, antiviral, and neuroprotective effects [43]. When compared to the literature findings for extracts of plants, the present results suggested that phenolics, flavonoids, and terpenoids alone or in combination may be the major contributors for the antioxidant, enzyme inhibitory, and neuroprotective activities.

## 4. Conclusions

Methanolic extract and its derived chloroform fraction of* A. alnifolia*,* I. indica,* and* O. obtusata* were characterized with respect to their inhibitory activity against AChE and BuChE and *α*-glucosidase enzymes and phytochemical profile. Concerning cholinesterases, the chloroform fractions displayed strong dual inhibitory potencies on AChE and BuChE. Similarly, the same fractions also disclosed high inhibitory abilities against *α*-glucosidase. The results of different radical scavenging assays indicate that most active fractions against target enzymes demonstrated greater antioxidant capacities also. Accordingly, the active chloroform fractions have also excelled neuroprotective activity against H_2_O_2_ induced oxidative stress in neuronal cells. The chemical prospecting of active fractions suggested that phenolic, flavonoid, and terpenoid contents alone or in combination are responsible for their strong anti-DM and anti-AD activities. Finally, this study is the first report that shows the fact that chloroform fraction of methanolic extract of* A. alnifolia*,* P. indica,* and* O. obtusata* has multifunctional therapeutic potential for the treatment of both DM and AD. There is indeed a pressing need to make new plant derived effective and safe bioactive molecules available; thus,* A. alnifolia*,* P. indica,* and* O. obtusata* may be a great natural source for the establishment of new agents in combating age-related neurodegenerative diseases like DM and AD combinedly.

## Supplementary Material

Figure S1: Steady-state inhibition of AChE (A), BuChE (B), alpha–Glucosidase (C) by most active fraction (PIC) from Pevetta indica. (Left) Lineweaver Burk plots; (right) secondary plots of the Lineweaver Burk plots.
Figure S2: Steady-state inhibition of AChE (A), BChE (B), alpha–Glucosidase (C)) by most active fraction (OOC) from Ochna obtusata. (Left) Lineweaver Burk plots; (right) secondary plots of the Lineweaver Burk plots.
Figure S3a: Inhibitory study of Acalifa alnifolia towards AChE. Figure S3b: Inhibitory study of Acalifa alnifolia towards BuChE. Figure S3c: Inhibitory study of Acalifa alnifolia towards alpha-glucosidase.
Figure S4a: Inhibitory study of Pavetta indica towards AChE. Figure S4b: Inhibitory study of Pavetta indica towards BuChE. Figure S4c: Inhibitory study of Pavetta indica towards alpha-glucosidase.
Figure S5a: Inhibitory study of Ochna obtusata towards AChE. Figure S5b: Inhibitory study of Ochna obtusata towards BuChE. Figure S5c: Inhibitory study of Ochna obtusata towards alpha-glucosidase.

## Figures and Tables

**Table 1:** Details of Indian medicinal plants used in the present study and their traditional uses.

Plant name	Local name	Family	Traditional uses	Reported activities
*A. alnifolia*	Mirapa kuppinta	Euphorbiaceae	Root: diarrhoea, indigestion, and insect bite. Leaves: earache, emetic, menorrhagia, migraine, scabies, anthelmintic, and diabetes [18].	Leaves have biolarvicidal and pupicidal activity [18] and are antioxidant [19].

*P. indica*	Papidi	Rubiaceae	Leaves: piles, haemorrhoidal pains, fever, and diarrhoea. Root: diuretic, purgative, tonic, and urinary disorders [18]. This is also used to relieve haemorrhoidal pain, as a lotion for nose, as analgesic, as antipyretic, as appetizer, for the ulceration of mouth [20, 21].	Inhibit corrosion of mild steel in acid media [22]. Antibacterial, antiviral, and antimalarial activities [23].

*O. obtusata*	Sunari	Ochnaceae	Leaves: lumbago, ulcers, asthma, and bronchitis. Root: menstrual complaints, constipation, asthma, and poisonous bites. Bark: digestive, tonic [18].	Ulcer, asthma, and bronchitis [24].

**Table 2:** Percentage yield of methanolic extracts and derived fractions of *A. alnifolia*,* P. indica,* and *O. obtusata*.

Plant	Part used	% of yield per 100 g of dry weight			
		90% MeOH	CHCl_3_	*n*-BuOH	H_2_O
*A. alnifolia*	Aerial parts	17.68	5.28	2.03	9.44
*P. indica*	Aerial parts	15.34	5.11	3.19	6.7
*O. obtusata*	Leaves	16.97	4.2	3.92	7.98

**Table 3:** IC_50_ values of 90% methanolic extracts and their derived fractions from titled plants for AChE, BuChE, and *α*-Glc inhibition assays.

Plant	Extract	IC_50_ values (*µ*g/mL)		
		AChE	BuChE	*α*-Glc
*A. alnifolia*	90% MeOH	59.22 ± 2.15	41.74 ± 1.27	45.24 ± 4.01
	CHCl_3_	32.88 ± 3.006	33.62 ± 2.83	41.86 ± 1.31
	*n*-BuOH	257.47 ± 11.89	144.27 ± 12.56	264.41 ± 29.9
	H_2_O	64.8 ± 3.22	61.6 ± 5.3	286.4 ± 19.99

*P. indica*	90% MeOH	17.77 ± 3.17	20.22 ± 2.8	42.76 ± 2.0
	CHCl_3_	52.09 ± 2.5	38.97 ± 8.1	35.29 ± 1.6
	*n*-BuOH	60.12 ± 0.84	74.67 ± 2.7	92.99 ± 2.8
	H_2_O	100.37 ± 1.99	355.95 ± 4.7	50.27 ± 2.3

*O. obtusata*	90% MeOH	82.16 ± 2.55	47.48 ± 18.27	28.7 ± 1.9
	CHCl_3_	25.7 ± 0.3	19.83 ± 1.28	39.3 ± 1.6
	*n*-BuOH	174.38 ± 27.005	233.28 ± 13.26	87.07 ± 4.9
	H_2_O	369.15 ± 8.6	332.28 ± 18.17	97.35 ± 9.7

Galantamine	—	0.77 ± 0.09	8.1 ± 0.02	—

Acarbose	—	—	—	117.20 ± 0.017

**Table 4:** Kinetic study of most active chloroform fractions AAC, PIC, and OOC on AChE, BuChE, and *α*-Glc enzyme inhibition.

Plant	Enzymes	Type of inhibition	Inhibition constant (*K*_*i*_) (*µ*g/mL)	
			*K*_*i*1_	*K*_*i*2_
*A. alnifolia*	AChE	Mixed	51.84	177.5
	BuChE	Mixed	70.51	78.4
	*α*-Glc	Mixed	42.67	150.9

*P. indica*	AChE	Mixed	56.35	85.9
	BuChE	Mixed	37.9	89.01
	*α*-Glc	Mixed	97.71	169.0

*O. obtusata*	AChE	Mixed	51.84	135.01
	BuChE	Mixed	92.33	177.4
	*α*-Glc	Mixed	50.98	73.4

**Table 5:** ABTS and DPPH free radical scavenging activity of CHCl_3_ fractions.

Plant	ABTS *µ*mol TE/g	DPPH *µ*mol AAE/g
*A. alnifolia*	143.39 ± 2.17	127.18 ± 1.28
*P. indica*	150.29 ± 1.58	204.4 ± 2.21
*O. obtusata*	171.006 ± 3.32	183.96 ± 5.29

**Table 6:** Quantitative phytochemical analysis of the active fractions.

S. number	Plant	TPC mg GAE/g	TFC mg RE/g	TTC mg CE/g	TTRC mg LE/g	TAC mg AE/g
(1)	*A. alnifolia*	140.55 ± 1.99	30.09 ± 2.4	5.004 ± 0.104	16.8 ± 1.46	0.68 ± 0.04
(2)	*P. indica*	19.53 ± 0.98	99.52 ± 4.6	1.6 ± 0.16	24.9 ± 0.28	16.85 ± 1.24
(3)	*O. obtusata*	174.9 ± 7.6	260.93 ± 22.4	14.658 ± 0.045	358.7 ± 0.9	6.18 ± 0.55

TPC: total phenolic content, TFC: total flavonoid content, TTRC: total terpenoid content, TAC: total alkaloid content, GAE: gallic acid equivalents, RE: rutin equivalents, CE: catechin equivalents, LE: linalool equivalents, and AE: atropin equivalents.
