# Supplementary material for: In Vitro Screening of Three Indian Medicinal Plants for Their Phytochemicals, Anticholinesterase, Antiglucosidase, Antioxidant, and Neuroprotective Effects
Source: Biomed Res Int. 2017 Oct 24;2017:5140506. doi: 10.1155/2017/5140506 (PMC5674485; doi:10.1155/2017/5140506)
Supplement: Supplementary file 1 — Figure S1: Steady-state inhibition of AChE (A), BuChE (B), alpha–Glucosidase (C) by most active fraction (PIC) from Pevetta indica. (Left) Lineweaver Burk plots; (right) secondary plots of the Lineweaver Burk plots. Figure S2: Steady-state inhibition of AChE (A), BChE (B), alpha–Glucosidase (C)) by most active fraction (OOC) from Ochna obtusata. (Left) Lineweaver Burk plots; (right) secondary plots of the Lineweaver Burk plots. Figure S3a: Inhibitory study of Acalifa alnifolia towards AChE. Figure S3b: Inhibitory study of Acalifa alnifolia towards BuChE. Figure S3c: Inhibitory study of Acalifa alnifolia towards alpha-glucosidase. Figure S4a: Inhibitory study of Pavetta indica towards AChE. Figure S4b: Inhibitory study of Pavetta indica towards BuChE. Figure S4c: Inhibitory study of Pavetta indica towards alpha-glucosidase. Figure S5a: Inhibitory study of Ochna obtusata towards AChE. Figure S5b: Inhibitory study of Ochna obtusata towards BuChE. Figure S5c: Inhibitory study of Ochna obtusata towards alpha-glucosidase. [file 5140506.f1.docx]

Kinetic Study of Pevetta indica on AChE, BuChE and α-Glucosidase

|  |  |  |
| --- | --- | --- |
|  |  |  |
|  |  |  |

Figure S1: Steady-state inhibition of AChE (A), BuChE (B), α–Glucosidase (C) by most active fraction (PIC) from Pevetta indica. (Left) Lineweaver Burk plots; (right) secondary plots of the Lineweaver Burk plots

Kinetic Study of *Ochna obtusata* on AChE, BuChE,and α-Glucosidase

|  |  |  |
| --- | --- | --- |
|  |  |  |
|  |  |  |

Figure S2: Steady-state inhibition of AChE (A), BChE (B), α–Glucosidase (C)) by most active fraction (OOC) from *Ochna obtusata.* (Left) Lineweaver Burk plots; (right) secondary plots of the Lineweaver Burk plots

Figure S3a: Inhibitory study of *Acalifa alnifolia* towards AChE.

| Con.c MeOH CHCl_3_ n-BuOH H_2_O   \| 15 \| 25.64 \| 38.5 \| 16.9 \| 24.87 \| \| --- \| --- \| --- \| --- \| --- \| \| 30 \| 40.68 \| 42.1 \| 25.36 \| 39.47 \| \| 90 \| 57.61 \| 65 \| 32.89 \| 50.17 \| \| 150 \| 67.26 \| 87 \| 43.64 \| 63.64 \| |  |
| --- | --- | --- | --- | --- | --- | --- | --- | --- | --- | --- | --- | --- | --- | --- | --- | --- | --- | --- | --- | --- | --- |
| Con.c MeOH CHCl_3_ n-BuOH H_2_O   \| 15 \| 21.36 \| 41.5 \| 20.694 \| 21.24 \| \| --- \| --- \| --- \| --- \| --- \| \| 30 \| 45.29 \| 49.64 \| 27.98 \| 40.36 \| \| 90 \| 51.31 \| 68.35 \| 35.18 \| 52.28 \| \| 150 \| 65.61 \| 80.69 \| 50.19 \| 69.64 \| |  |
| Con.c MeOH CHCl_3_ n-BuOH H_2_O   \| 15 \| 20.36 \| 35.69 \| 8.94 \| 10.26 \| \| --- \| --- \| --- \| --- \| --- \| \| 30 \| 41.26 \| 44.67 \| 26.19 \| 25.026 \| \| 90 \| 57.32 \| 61.36 \| 37.29 \| 40.26 \| \| 150 \| 68.94 \| 77.39 \| 45.49 \| 45.69 \| \|  \|  \|  \|  \|  \| |  |

Figure S3b: Inhibitory study of *Acalifa alnifolia* towards BuChE.

| Con.c MeOH CHCl_3_ n-BuOH H_2_O   \| 15 \| 30.25 \| 34 \| 18.96 \| 31.13 \| \| --- \| --- \| --- \| --- \| --- \| \| 30 \| 42.25 \| 59 \| 25.6 \| 42.25 \| \| 90 \| 66.29 \| 68 \| 41.05 \| 51.26 \| \| 150 \| 80.58 \| 75 \| 50.28 \| 64.98 \| |  |
| --- | --- | --- | --- | --- | --- | --- | --- | --- | --- | --- | --- | --- | --- | --- | --- | --- | --- | --- | --- | --- | --- |
| Con.c MeOH CHCl_3_  n-BuOH H_2_O   \| 15 \| 27.9 \| 30.36 \| 20.59 \| 29.25 \| \| --- \| --- \| --- \| --- \| --- \| \| 30 \| 40.6 \| 55.28 \| 30.69 \| 40.18 \| \| 90 \| 65.18 \| 64.27 \| 39.58 \| 55.69 \| \| 150 \| 83.69 \| 76.64 \| 55.69 \| 70.29 \| |  |
| Con.c MeOH CHCl_3_  n-BuOH H_2_O   \| 15 \| 25.69 \| 24.01 \| 32.58 \| 27.12 \| \| --- \| --- \| --- \| --- \| --- \| \| 30 \| 35.29 \| 39.27 \| 44.59 \| 48.59 \| \| 90 \| 41.36 \| 60.21 \| 50.19 \| 60.21 \| \| 150 \| 56.36 \| 88.89 \| 75.16 \| 72.16 \| |  |

Figure S3c: Inhibitory study of *Acalifa alnifolia* towards α-glucosidase.

| Con.c MeOH CHCl_3_ n-BuOH H_2_O   \| 15 \| 35.35 \| 30.35 \| 15.19 \| 21.25 \| \| --- \| --- \| --- \| --- \| --- \| \| 30 \| 50.13 \| 46.13 \| 25.18 \| 37.68 \| \| 90 \| 65.35 \| 60.35 \| 31.29 \| 51.28 \| \| 150 \| 77.16 \| 77.16 \| 45.28 \| 62.28 \| \|  \|  \|  \|  \|  \| \|  \|  \|  \|  \|  \| |  |
| --- | --- | --- | --- | --- | --- | --- | --- | --- | --- | --- | --- | --- | --- | --- | --- | --- | --- | --- | --- | --- | --- | --- | --- | --- | --- | --- | --- | --- | --- | --- | --- |
| Con.c MeOH CHCl_3_ n-BuOH H_2_O   \| 15 \| 32.26 \| 31.25 \| 21.35 \| 19.27 \| \| --- \| --- \| --- \| --- \| --- \| \| 30 \| 45.21 \| 50.26 \| 26.59 \| 24.29 \| \| 90 \| 57.29 \| 62.19 \| 33.26 \| 49.26 \| \| 150 \| 79.31 \| 70.29 \| 47.16 \| 60.25 \| \|  \|  \|  \|  \|  \| \|  \|  \|  \|  \|  \| |  |
| Con.c MeOH CHCl_3_ n-BuOH H_2_O   \| 15 \| 27.29 \| 27.12 \| 20.15 \| 22.25 \| \| --- \| --- \| --- \| --- \| --- \| \| 30 \| 40.19 \| 48.59 \| 27.16 \| 35.28 \| \| 90 \| 55.27 \| 60.21 \| 38.16 \| 50.16 \| \| 150 \| 72.16 \| 72.16 \| 48.26 \| 61.26 \| \|  \|  \|  \|  \|  \| |  |

Figure S4a: Inhibitory study of *Pavetta indica* towards AChE.

| Con.c MeOH CHCl_3_ n-BuOH H_2_O   \| 15 \| 52.26 \| 25.59 \| 25.16 \| 17.36 \| \| --- \| --- \| --- \| --- \| --- \| \| 30 \| 58.69 \| 36.69 \| 31.25 \| 30.25 \| \| 90 \| 71.26 \| 57.69 \| 47.11 \| 42.69 \| \| 150 \| 81.26 \| 71.29 \| 58.49 \| 60.26 \| |  |
| --- | --- | --- | --- | --- | --- | --- | --- | --- | --- | --- | --- | --- | --- | --- | --- | --- | --- | --- | --- | --- | --- |
| Con.c MeOH CHCl_3_ n-BuOH H_2_O   \| 15 \| 42.26 \| 27.59 \| 22.15 \| 16.29 \| \| --- \| --- \| --- \| --- \| --- \| \| 30 \| 55.29 \| 39.58 \| 34.97 \| 31.26 \| \| 90 \| 67.369 \| 58.46 \| 45.69 \| 44.19 \| \| 150 \| 77.49 \| 78.59 \| 60.59 \| 58.59 \| |  |
| Con.c MeOH CHCl_3_ n-BuOH H_2_O   \| 15 \| 49.58 \| 21.25 \| 20.15 \| 18.29 \| \| --- \| --- \| --- \| --- \| --- \| \| 30 \| 60.36 \| 35.27 \| 35.29 \| 32.26 \| \| 90 \| 74.26 \| 60.28 \| 50.27 \| 45.29 \| \| 150 \| 85.59 \| 75.29 \| 61.29 \| 59.68 \| \|  \|  \|  \|  \|  \| |  |

Figure S4b: Inhibitory study of *Pavetta indica* towards BuChE.

| Con.c MeOH CHCl_3_ n-BuOH H_2_O   \| 15 \| 47.3 \| 22.19 \| 21.02 \| 11.07 \| \| --- \| --- \| --- \| --- \| --- \| \| 30 \| 60.26 \| 30.26 \| 37.89 \| 17.49 \| \| 90 \| 71.26 \| 42.29 \| 55.26 \| 27.18 \| \| 150 \| 88.59 \| 51.29 \| 62.37 \| 44.6 \| \|  \|  \|  \|  \|  \| \|  \|  \|  \|  \|  \| |  |
| --- | --- | --- | --- | --- | --- | --- | --- | --- | --- | --- | --- | --- | --- | --- | --- | --- | --- | --- | --- | --- | --- | --- | --- | --- | --- | --- | --- | --- | --- | --- | --- |
| Con.c MeOH CHCl_3_ n-BuOH H_2_O   \| 15 \| 45.26 \| 20.16 \| 20.14 \| 9.58 \| \| --- \| --- \| --- \| --- \| --- \| \| 30 \| 57.98 \| 27.98 \| 33.27 \| 15.27 \| \| 90 \| 69.69 \| 41.16 \| 51.36 \| 30.28 \| \| 150 \| 82.16 \| 55.29 \| 63.27 \| 37.19 \| \|  \|  \|  \|  \|  \| |  |
| Con.c MeOH CHCl_3_ n-BuOH H_2_O   \| 15 \| 49.69 \| 19.59 \| 18.69 \| 10.02 \| \| --- \| --- \| --- \| --- \| --- \| \| 30 \| 58.94 \| 31.26 \| 30.25 \| 14.16 \| \| 90 \| 70.18 \| 43.19 \| 50.14 \| 33.16 \| \| 150 \| 80.15 \| 50.29 \| 65.29 \| 39.46 \| \|  \|  \|  \|  \|  \| |  |

Figure S4c: Inhibitory study of *Pavetta indica* towards α-glucosidase.

| Con.c MeOH CHCl_3_ n-BuOH H_2_O   \| 15 \| 32.25 \| 38.26 \| 21.18 \| 30.15 \| \| --- \| --- \| --- \| --- \| --- \| \| 30 \| 45.26 \| 50.26 \| 30.25 \| 42.25 \| \| 90 \| 66.569 \| 64.19 \| 48.59 \| 53.26 \| \| 150 \| 78.29 \| 72.19 \| 57.89 \| 68.39 \| \|  \|  \|  \|  \|  \| |  |
| --- | --- | --- | --- | --- | --- | --- | --- | --- | --- | --- | --- | --- | --- | --- | --- | --- | --- | --- | --- | --- | --- | --- | --- | --- | --- | --- |
| Con.c MeOH CHCl_3_ n-BuOH H_2_O   \| 15 \| 30.25 \| 32.02 \| 20.12 \| 37.26 \| \| --- \| --- \| --- \| --- \| --- \| \| 30 \| 43.12 \| 51.05 \| 29.59 \| 44.15 \| \| 90 \| 65.13 \| 61.02 \| 45.79 \| 57.69 \| \| 150 \| 76.14 \| 73.28 \| 60.25 \| 64.12 \| \|  \|  \|  \|  \|  \| |  |
| Con.c MeOH CHCl_3_ n-BuOH H_2_O   \| 15 \| 31.15 \| 33.02 \| 25.14 \| 33.28 \| \| --- \| --- \| --- \| --- \| --- \| \| 30 \| 45.17 \| 55.15 \| 34.58 \| 47.49 \| \| 90 \| 61.12 \| 60.58 \| 46.26 \| 60.15 \| \| 150 \| 72.25 \| 70.15 \| 61.04 \| 70.18 \| |  |

Figure S5a: Inhibitory study of *Ochna obtusata* towards AChE.

| Con.c MeOH CHCl_3_ n-BuOH H_2_O   \| 15 \| 49.68 \| 41.01 \| 16.76 \| 8.76 \| \| --- \| --- \| --- \| --- \| --- \| \| 30 \| 57.15 \| 54.19 \| 24.19 \| 19.49 \| \| 90 \| 69.25 \| 67.46 \| 38.58 \| 22.19 \| \| 150 \| 78.25 \| 81.07 \| 45.28 \| 44.28 \| \|  \|  \|  \|  \|  \| \|  \|  \|  \|  \|  \| |  |
| --- | --- | --- | --- | --- | --- | --- | --- | --- | --- | --- | --- | --- | --- | --- | --- | --- | --- | --- | --- | --- | --- | --- | --- | --- | --- | --- | --- | --- | --- | --- | --- |
| Con.c MeOH CHCl_3_ n-BuOH H_2_O   \| 15 \| 48.19 \| 40.29 \| 20.18 \| 4.69 \| \| --- \| --- \| --- \| --- \| --- \| \| 30 \| 58.79 \| 55.18 \| 31.15 \| 20.19 \| \| 90 \| 72.49 \| 68.17 \| 40.25 \| 28.97 \| \| 150 \| 81.26 \| 80.79 \| 49.87 \| 38.49 \| \|  \|  \|  \|  \|  \| |  |
| Con.c MeOH CHCl_3_ n-BuOH H_2_O   \| 15 \| 46.28 \| 38.48 \| 18.57 \| 9.57 \| \| --- \| --- \| --- \| --- \| --- \| \| 30 \| 60.25 \| 56.17 \| 32.57 \| 22.15 \| \| 90 \| 77.49 \| 67.49 \| 44.58 \| 37.19 \| \| 150 \| 86.97 \| 85.59 \| 51.27 \| 47.19 \| \|  \|  \|  \|  \|  \| \|  \|  \|  \|  \|  \| |  |

Figure S5b: Inhibitory study of *Ochna obtusata* towards BuChE.

| Con.c MeOH CHCl_3_ n-BuOH H_2_O   \| 15 \| 48.36 \| 45.28 \| 13.67 \| 11.14 \| \| --- \| --- \| --- \| --- \| --- \| \| 30 \| 62.19 \| 57.26 \| 21.15 \| 16.89 \| \| 90 \| 80.19 \| 62.18 \| 46.59 \| 25.26 \| \| 150 \| 91.59 \| 80.26 \| 61.23 \| 37.25 \| |  |
| --- | --- | --- | --- | --- | --- | --- | --- | --- | --- | --- | --- | --- | --- | --- | --- | --- | --- | --- | --- | --- | --- |
| Con.c MeOH CHCl_3_ n-BuOH H_2_O   \| 15 \| 48.18 \| 50.29 \| 19.15 \| 10.25 \| \| --- \| --- \| --- \| --- \| --- \| \| 30 \| 58.18 \| 64.19 \| 27.16 \| 15.29 \| \| 90 \| 66.18 \| 78.49 \| 45.69 \| 24.59 \| \| 150 \| 78.95 \| 89.46 \| 60.25 \| 31.26 \| \|  \|  \|  \|  \|  \| |  |
| Con.c MeOH CHCl_3_ n-BuOH H_2_O   \| 15 \| 45.26 \| 51.12 \| 20.26 \| 11.25 \| \| --- \| --- \| --- \| --- \| --- \| \| 30 \| 55.59 \| 65.19 \| 31.25 \| 16.59 \| \| 90 \| 70.25 \| 74.56 \| 47.16 \| 29.15 \| \| 150 \| 81.25 \| 84.59 \| 66.28 \| 37.69 \| \|  \|  \|  \|  \|  \| |  |

Figure S5c: Inhibitory study of *Ochna obtusata* towards α-glucosidase.

| Con.c MeOH CHCl_3_ n-BuOH H_2_O   \| 15 \| 41.25 \| 32.17 \| 19.69 \| 15.69 \| \| --- \| --- \| --- \| --- \| --- \| \| 30 \| 50.18 \| 45.26 \| 30.16 \| 30.21 \| \| 90 \| 70.16 \| 62.21 \| 46.69 \| 45.26 \| \| 150 \| 81.26 \| 71.12 \| 58.98 \| 55.28 \| \|  \|  \|  \|  \|  \| |  |
| --- | --- | --- | --- | --- | --- | --- | --- | --- | --- | --- | --- | --- | --- | --- | --- | --- | --- | --- | --- | --- | --- | --- | --- | --- | --- | --- |
| Con.c MeOH CHCl_3_ n-BuOH H_2_O   \| 15 \| 37.16 \| 31.25 \| 21.02 \| 16.58 \| \| --- \| --- \| --- \| --- \| --- \| \| 30 \| 48.65 \| 43.69 \| 35.26 \| 32.15 \| \| 90 \| 65.59 \| 65.21 \| 49.65 \| 44.16 \| \| 150 \| 80.16 \| 80.15 \| 60.28 \| 58.69 \| |  |
| Con.c MeOH CHCl_3_ n-BuOH H_2_O   \| 15 \| 40.16 \| 29.21 \| 22.12 \| 20.15 \| \| --- \| --- \| --- \| --- \| --- \| \| 30 \| 51.61 \| 47.69 \| 34.69 \| 40.16 \| \| 90 \| 69.94 \| 70.12 \| 50.26 \| 50.16 \| \| 150 \| 84.61 \| 79.021 \| 61.02 \| 60.28 \| \|  \|  \|  \|  \|  \| \|  \|  \|  \|  \|  \| \|  \|  \|  \|  \|  \| |  |
